# Association between IL-6 production in synovial explants from rheumatoid arthritis patients and clinical and imaging response to biologic treatment: A pilot study

**DOI:** 10.1371/journal.pone.0197001

**Published:** 2018-05-22

**Authors:** Martin Andersen, Mikael Boesen, Karen Ellegaard, Kalle Söderström, Niels H. Søe, Pieter Spee, Ulrik G. W. Mørch, Søren Torp-Pedersen, Else M. Bartels, Bente Danneskiold-Samsøe, Lars Karlsson, Henning Bliddal

**Affiliations:** 1 The Parker Institute, Department of Rheumatology, Copenhagen University Hospital, Bispebjerg and Frederiksberg, Copenhagen, Denmark; 2 Translational Immunology, Biopharmaceutical Research Unit, Måløv, Novo Nordisk, Denmark; 3 Department of Radiology, Copenhagen University Hospital, Bispebjerg and Frederiksberg, Copenhagen, Denmark; 4 Department of Orthopaedics, Section of Hand Surgery, Gentofte University Hospital, Hellerup, Denmark; 5 Biomarkers, Novo Nordisk, Søborg, Denmark; Keio University, JAPAN

## Abstract

**Introduction:**

The need for biomarkers which can predict disease course and treatment response in rheumatoid arthritis (RA) is evident. We explored whether clinical and imaging responses to biologic disease modifying anti-rheumatic drug treatment (bDMARD) were associated with the individual’s mediator production in explants obtained at baseline.

**Methods:**

RA Patients were evaluated by disease activity score 28 joint C-reactive protein (DAS 28-)), colour Doppler ultrasound (CDUS) and 3 Tesla RA magnetic resonance imaging scores (RAMRIS). Explants were established from synovectomies from a needle arthroscopic procedure prior to initiation of bDMARD. Explants were incubated with the bDMARD in question, and the productions of interleukin-6 (IL-6), monocyte chemo-attractive protein-1 (MCP-1) and macrophage inflammatory protein-1-beta (MIP-1b) were measured by multiplex immunoassays. The changes in clinical and imaging variables following a minimum of 3 months bDMARD treatment were compared to the baseline explant results. Mixed models and Spearman’s rank correlations were performed. P-values below 0.05 were considered statistically significant.

**Results:**

16 patients were included. IL-6 production in bDMARD-treated explants was significantly higher among clinical non-responders compared to responders (P = 0.04), and a lack of suppression of IL-6 by the bDMARDS correlated to a high DAS-28 (ρ = 0.57, P = 0.03), CDUS (ρ = 0.53, P = 0.04) and bone marrow oedema (ρ = 0.56, P = 0.03) at follow-up. No clinical association was found with explant MCP-1 production. MIP-1b could not be assessed due to a large number of samples below the detection limit.

**Conclusions:**

Synovial explants appear to deliver a disease-relevant output testing which when carried out in advance of bDMARD treatment can potentially pave the road for a more patient tailored treatment approach with better treatment effects.

## Introduction

Predicting response to treatment and achieving disease control without progressive joint destruction are among the greatest challenges in rheumatoid arthritis (RA). Joint destruction is driven by an inflammatory process encompassing numerous cell types, and leading to cartilage and bone damage by release of metalloproteases, as well as an activation of chondrocytes and osteoclasts[1,2]

With biologic disease modifying anti-rheumatic drugs (bDMARDs) emerging as a treatment option more than 20 years ago, a paradigm shift happened in RA treatment. However, it has become clear that only around 15 percent of RA patients achieve disease remission with bDMARDs [3–7]. Drug adherence is also short considering that potentially life-long treatment is required[3]. Switch to another bDMARD due to adverse events or treatment failure is common, and the choice of both first and second bDMARD is ruled by tradition rather, and the guidelines are not well-defined[8]. The increasing number of bDMARD options, and the unmet treatment challenges, warrant methods for testing drug efficacy at patient level. Studies have reported that changes in inflammatory markers such as interleukin 6 (IL-6) are associated with the clinical response to treatment [9,10]. However, baseline levels of biomarkers, which can be used for screening of RA patients with regards to choice of bDMARD have not yet been presented.

A possible approach to a patient-tailored treatment strategy could be explored using cultures of synovial tissue. Previous studies on explants obtained from RA patients undergoing arthroplasty, have demonstrated the cultures’ capacity to produce key inflammatory mediators involved in RA pathology. The production of these mediators can be modulated by addition of different bDMARDs or other immuno-modulatory compounds[11–15]. We recently demonstrated that synovial explants produce IL-6, monocyte chemo-attractant protein 1 (MCP-1) and macrophage inflammatory protein 1 beta (MIP-1b), and that this production was associated with colour Doppler ultrasound (CDUS) activity, magnetic resonance imaging (MRI) findings of synovitis, bone marrow oedema (BME), and erosions, using the RA MRI score (RAMRIS) and disease activity score 28 joints C-reactive protein (DAS-28) [16].

The aim of this study was to explore whether *in vitro* effects of a bDMARD on the individual’s baseline RA synovial explants were associated with the *in vivo* treatment response to the same bDMARD, both clinically and by imaging.

## Patients and methods

### Patients

The study period took place between May 2010 and October 2013. Study participants (N = 20) were recruited from a larger cohort of RA patients[16]. Inclusion criteria were as previously described; RA patients opted for bDMARD therapy with active arthritis involving hand joints as defined by synovial hypertrophy on ultrasound. Baseline and follow up evaluation included DAS28_CRP_, CDUS and 3 Tesla MRI of the joints opted for synovectomy. Within 24 hours after baseline examination, patients had a synovectomy performed of up to two joints on the same hand.

Patients were retested at follow-up after a minimum of three months of treatment, and European Legue Against Rheumatism (EULAR) response was determined[17,18]. Patients were excluded from the present study if a local steroid injection was given in the synovectomised joint during the follow-up period, or if daily steroid consumption exceeded 10 mg. Other reasons for exclusion were skin changes over the target joint, allergy to local anaesthetics, and anti-coagulatory treatment that could not be paused for 48 hours pre-surgery. Patient examination and imaging procedures were performed at the Departments of Rheumatology and Radiology Bispebjerg & Frederiksberg Hospital, Denmark. The study was approved by the Health Research Ethics Committee of the Capital Region of Denmark (study number No. H-4-2009-117), and signed informed consent was obtained from each patient.

### Procedures

The needle arthroscopic procedure was carried out at The Department of Orthopaedics, Section of Hand Surgery, Gentofte Hospital, approximately 24 hours after recording of baseline data. Briefly, synovectomies were performed using a 1.9 mm Karl-Storz arthroscope with a two portal technique ensuring that the surgeon (NS) had full visual control over the anatomical origin of the synovectomy material. Each patient could have up to two joints synovectomised, and up to six joint positions from the wrist and three joint positions from the metacarpo-phalangeal (MCP) joint. Mapping of synovial tissue with the corresponding anatomical sites on imaging was secured by the surgeon being guided by the CDUS description. S1 Table offers an overview of the anatomical landmarks of the synovectomy positions in the wrist.

### Imaging modalities

#### Ultrasound

The evaluation was performed by two experienced ultrasound specialists (KE or STP) using a GE Loqic E9 (Milwaukee, Wisconsin, USA) with a15 MHz centre frequency linear array matrix transducer. Doppler pre-set was adjusted for maximum sensitivity for low flow (pulse repetition frequency of 0.4 kHz, lowest wall filter on 45 Hz, and 7.5 MHz Doppler frequency), with Doppler gain just below noise level. This pre-set remained unchanged throughout the study period, and scanning positions were standardized according to anatomical landmarks. The ratio of colour pixels per unit of gray scale pixel count (the colour fraction—CF) in the systole, defined as CF_max_, was used as the outcome measure for colour Doppler activity. The pixel count was calculated by the use of an automated imaging processing program, ImagePro^™^[19]. All patients had wrists, MCP and PIP joints scanned from dorso-lateral positions only, to identify the up to two joints with most activity which would be selected for synovectomy. All examinations were performed at room temperature and around 10 AM. Smoking or use of nicotine substitutions 12 hours prior to the examination was not permitted.

#### MRI

Patients were examined in a supine position with the hands along the side of the body in a 3 Tesla MRI scanner (Siemens, Verio^®^, Erlangen Germany) using a 16-channel cardiac coil covering the target hand. Coronal and axial short tau inverted recovery (STIR) and pre-/post-contrast T1 weighed sequences were used for the RAMRIS scores of synovitis, BME and erosions[20]. The RAMRIS synovitis score ranges from no, low, moderate, and severe synovitis (0–3) based on subjective evaluation criteria. Similarly the RAMRIS BME component was evaluated from 0–3 in each bone of the wrist and 2^nd^-5^th^ MCP joint, where each step corresponded to a 33% increase in BME. The RAMRIS erosion score ranged from 0–10, each step corresponding to 10% increments in bone area eroded in the anatomy of interest. The anatomical site of synovectomy was mapped to the same area on the MRI and ultrasound images.

Imaging scores were averaged according to the anatomic location of the synovectomy, or if synovectomy positions had been pooled. MRI was performed by an experienced radiologist (MB) who was blinded to all patient characteristics and ultrasound data.

### Outcome measures for the bDMARD cohort

Associations of EULAR responses, with mean fold changes in synovial mediator production from baseline to follow-up, were chosen as the primary outcome. As secondary outcomes the changes in DAS-28, ultrasound and MRI scores were correlated with response to bDMARD treatment at a joint level. Changes in DAS-28, ultrasound colour Doppler (CDUS) and MRI parameters were defined as the difference from baseline to follow-up.

### Synovial explant assay

**Fig 1:**
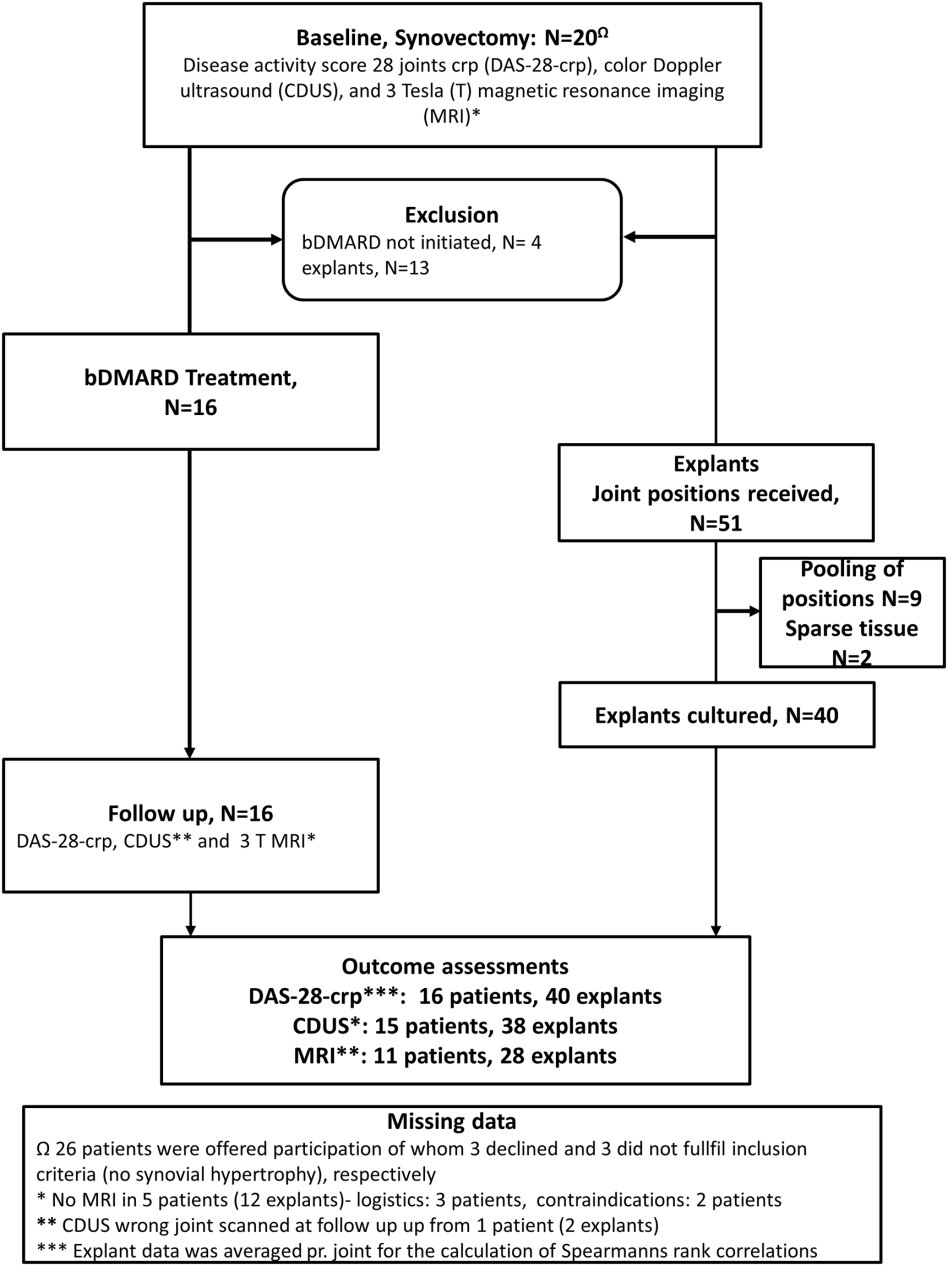
Flowchart outlining flow of patients, number of joints synovectomised and number of established synovial cultures. Rheumatoid arthritis patients with clinically suspected active arthritis involving the hand joints and opted for bDMARD treatment were evaluated by Doppler ultrasound for study participation. Explants were only included from patients, who were initiated in bDMARD treatment. bDMARD = biologic disease modifying anti-rheumatic treatment; CDUS = colour Doppler ultrasound; DAS-28 = Disease activity score 28 joints c-reactive protein; MRI = magnetic resonance imaging; T = Tesla; Ω = 26 patients were offered participation of whom 3 declined and 3 did not fulfil inclusion criteria (no synovial hypertrophy), respectively. *At baseline, MRI was not performed in 5 patients due to logistics (N = 3) and contra-indications (N = 2). ** At follow-up, CDUS was missing in 1 patient due to logistics. *** At follow-up MRI was only performed in 11 patients due to logistics.

Synovial explants were established as previously described[16]. In brief, synovial explants were distributed at approximately 2 mg per well in 96 flat bottom culture plates containing bovine bone slices. Tissue was incubated for 72 hours at 37°C with 95% O_2_ and 5% CO_2_ with 200 μL RPMI 1640 containing 10% heat inactivated (HI) foetal bovine serum and 2% HI human serum, penicillin and streptomycin. As depicted in Fig 1, at 72 hours of culture 50% medium was replaced and the relevant bDMARD added in triplicates or quadruplets, depending on the amount of available tissue. Medium was, furthermore, replaced after 1 week of culture, and finally aspirated after 2 weeks where the explant culture system was terminated. Supernatants from 72 hours and 2 weeks medium replacement were stored at -80°C, until analysed. Commercially available bDMARDs and isotype controls (IgG1 light chains, Sigma^®^) were added at 10μg/ml and/or 50 μg/ml. Each explant culture setup contained untreated wells for detection of spontaneous cytokine production, bDMARD-treated tissue, and isotype controls.

MCP-1, IL-6 and MIP-1b measurements were carried out at the laboratories of Myriad RBM^™^, Texas, USA, as previously described [16]. Supernatants were added 200μg/ml HeteroBlock (Omega Biologicals, Bozeman, MT, USA) and diluted 100 fold to avoid heterophilic antibody interference. If cytokine concentration was below assay detection limit, the lowest detection limit value was assigned. Lowest detection limits were: 72pg/mL (IL-6), 240 pg/mL (MCP-1) and 366 pg/mL (MIP-1b). Myriad RBM^™^ was, apart from the diagnosis, blinded for all patient characteristics, including imaging data.

Changes in cytokine concentration from baseline (72 hours) to two weeks were calculated as a ratio: (2 weeks/72 h). Wells with baseline cytokine production less than 20% of the average cytokine concentration were excluded from further analysis, since it was judged that these wells would not represent overall synovial activity. In the case of sparse tissue, synovectomy material was pooled from neighbouring positions.

## Statistical analysis

Due to the exploratory nature of the study, sample size was based on feasibility. A study population of 20 patients was judged to be sufficient for both practical and ethical reasons.

For imaging outcome measures and EULAR responses, mixed linear models were applied for the statistical tests, since data were clustered within patients, thereby preventing double-counting errors with inflated standard errors. The mixed model analysis, associating clinical response with fold change in mediator explant mediator production, included three pre-specified covariates: baseline mediator levels, EULAR response and type of *in vitro* intervention. As previously described[16], parsimony in the statistical models for imaging outcome variables was achieved by omitting design variables from the model, if no statistical significance was determined (p>0.1). For model optimization purposes, square root, inverse and logarithmic transformations were applied to achieve an approximate Gaussian distribution of residuals. Clinical outcome measures (DAS-28) were analysed by Spearman’s rank correlations, averaging explant activity data from the various joint positions in each particular patient. Since Spearman’s rho estimates were considered important to the overall visual data interpretation, Spearman’s estimates were calculated in the same way for the imaging data. P-values < 0.05 were considered statistically significant.

## Results

### Clinical outcomes

As depicted in Fig 1, a total of 20 patients were opted for bDMARD. Out of these, 16 were initiated on bDMARD and included in the statistical analysis. These patients consisted primarily of seropositive women (65%) with high disease activity (median DAS-28 = 5.4, and median CRP = 27 mg/Ll) and long-standing disease (a median of 8.5 years) (Table 1). At baseline 15 patients received various DMARDs, mostly methotrexate in monotherapy, while one patient received 5 mg prednisolone as monotherapy. Five of the 16 patients were bDMARD failures (infliximab: N = 4, rituximab N = 1), and thus synovectomised during a pause prior to switching to another bDMARD. The other 11 patients were treated de novo with their bDMARD. In 5 patients, 10 mg prednisolone per day was given in combination with the conventional DMARD at baseline. Three patients withdrew from their other anti-rheumatic drug (Leflunomide, Methotrexate and prednisolone, respectively) and received bDMARD as monotherapy (Etanercept, Certulizumab and Tocilizumab, respectively) during the study period.

**Table 1:** Patient demographics and clinical characteristics.

Variables	Baseline (N = 16)	Follow up (N = 16)	Change
Female, no. (%)*	11 (65%)	-	-
Age, years	61.2 [44.9;67.8]	-	-
Disease duration, years	8.5 [5.8;14.0]	-	-
RF-positive, no. (%)*	14 (88%)	-	-
Anti-CCP-positive, no. (%)*	14 (88%)	-	-
DAS-28-CRP, score: 0–10	5.4 [3.5;5.7]	3.4 [2.8; 3.7]	-1.8 [-3.0;0.1]
C-reactive protein, mg/L	27 [5;38]	3.5 [1.0;6.5]	-13.5 [-34.5; 0.0]
VAS patient global, scale: 0–100	81 [69;86]	30 [19;66]	-22 [-54;-4]
Tender joint count, 28 joints	5.5 [2.5;9.0]	2.0 [1.0;4.0]	-3.0[-5.5;0.3]
Swollen joint count, 28 joints	6.0 [3.8;9.0]	3.0 [1.0;5.0]	-3.5[-8.0;-0.8]
Biologic DMARD, total (%)*	0 (0%)¥	16 (100%)	-
Abatacept	0	1	-
Certulizumab	0	3	-
Etanercept	0	6	-
Infliximab	0	1	-
Mabthera	0	2	-
RoActemra	0	3	-
Conventional DMARD			
MTX, no. (%)^•^^,^*	10 (63%)	10 (63%)^‡^	0
SZS, no. (%)*	1 (6%)	1 (6%)^‡^	0
LFU, no. (%)*	4 (25%)	2 (13%)^‡^	-2
Prednisolone therapy, 10 mg/day^∞^	6 (6%)	1 (6%)^‡^	-5

Overview of patient demographics at baseline and change at follow up. Patients were followed for a medium time of 10 months (IQR 7 to 11 months). At follow up patients had been receiving bDMARD for a medium time of 7 months (IQR 5 to 9 months). Values are median [Q1; Q3]. LFU = Leflunomide, MCP = Metacarpo-phalangeal joint, MTX = Methotrexate, no. = number, PIP = Proximal interphalangeal joint, SD = standard deviation, Q1 = 1st Quartile, Q3 = 3rd Quartile, SZS = Salazopyrin, VAS = Visual analogue scale.–data not reported.

*percentage of patients;

^‡^ in combination with bDMARD;

^¥^ 5 patients were previous bDMARD failures (4 infliximab; 1 rituximab)

^•^Six patients were in MTX monotherapy, 2 patients MTX+SZS, 2 patients in triple DMARD;

^∞^ At baseline 5 patients received prednisolone in combination with conventional DMARD, 1 patient received prednisolone monotherapy at 5 mg.

No patient was lost during the study period. Follow-up was after bDMARD treatment for a median of 7.0 months (IQR 6.8 to 11.3 months). The patients showed a median change in DAS28 of -1.7 (IQR: -3.1; 0.3), and a median CRP reduction of 14 mg/ml (IQR: -35; 0.0). At follow-up, 1 patient had withdrawn from the bDMARD due to non-response.

### Explant cultures

In the 16 patients, 51 joint positions were synovectomized. Due to sparse material in some positions, 40 explant cultures (38 cultures from wrists and two cultures from MCP joints) were established. On average 95 mg of wet weigh tissue was harvested from wrist-joints and 56 mg from MCP joints.

All explants cultures exhibited progressive cellular outgrowth throughout the 2 week culture period when examined under light microscope during harvest of supernatants. A detailed overview of the fold change in cytokine production, grouped by in vitro treatment and EULAR response, is found in the additional files, as additional file 2. Median fold increase of IL-6 and MCP-1 was generally increasing through the study period. MIP-1b production remained low and was discarded from data-analysis since 42% of samples remained under assay detection limit at two weeks in contrast to IL-6 (12%) and MCP-1 (15%). Two patients, one Rituximab treated and one treated with Cimzia had only data available from wells treated with50 μg/ml, which were included in the statistical analysis.

### Associations of EULAR response to changes in explant mediator production

**Fig 2:**
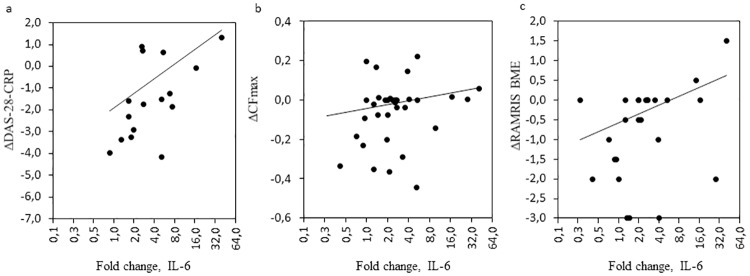
Scatter plots depicting fold changes in explant IL-6 production versus changes in DAS-28, CFmax and RAMRIS BME. Scatter plots depicting fold changes (2 week/72h) in IL-6 versus changes in DAS-28 (16 patients, 40 explants), CF_max_ (N = 15, 38 explants) and RAMRIS BME score (N = 11, 28 explants) among bDMARD-treated patients. Correlations were calculated using Spearman’s rank coefficients and P-values were calculated using mixed effects models for the imaging data A) ρ = 0.56, P = 0.03; B) ρ = 0.52, P = 0.04; C) ρ = 0.56, P = 0.03. bDMARD = *in vitro* added biologic Disease Modifying Anti-Rheumatic Drug; CFmax = maximal color fraction,; DAS-28 = Disease Activity Score of 28 joints including C reactive protein; IL-6 = interleukine 6; RAMRIS BME = Rheumatoid Arthritis Magnetic Resonance Imaging Score for Bone Marrow Oedema.

EULAR good responders had a significantly lower fold change in IL-6 of bDMARD-treated samples in contrast to non-responders (P = 0.04), with a mean fold difference of 3.45 (CL_95_ = 1.06; 11.25). IL-6 production of bDMARD-cultured samples was significantly lower than matched isotype controls in samples from good responders (P = 0.01), with a mean decrease of 45% (CL_95_ = 66%; 14%). The difference in IL-6 production was borderline significant with regards to spontaneous production and bDMARD (P = 0.06). No significant differences were seen with regards to *in vitro* effects among moderate responders or no responders, or with any groups and changes in MCP-1. For further details please consult Fig 2 and S2 Table. For model optimization purposes, one data point out of 236 was excluded from the analysis (Cooks distance = 0.6).

**Fig 3:**
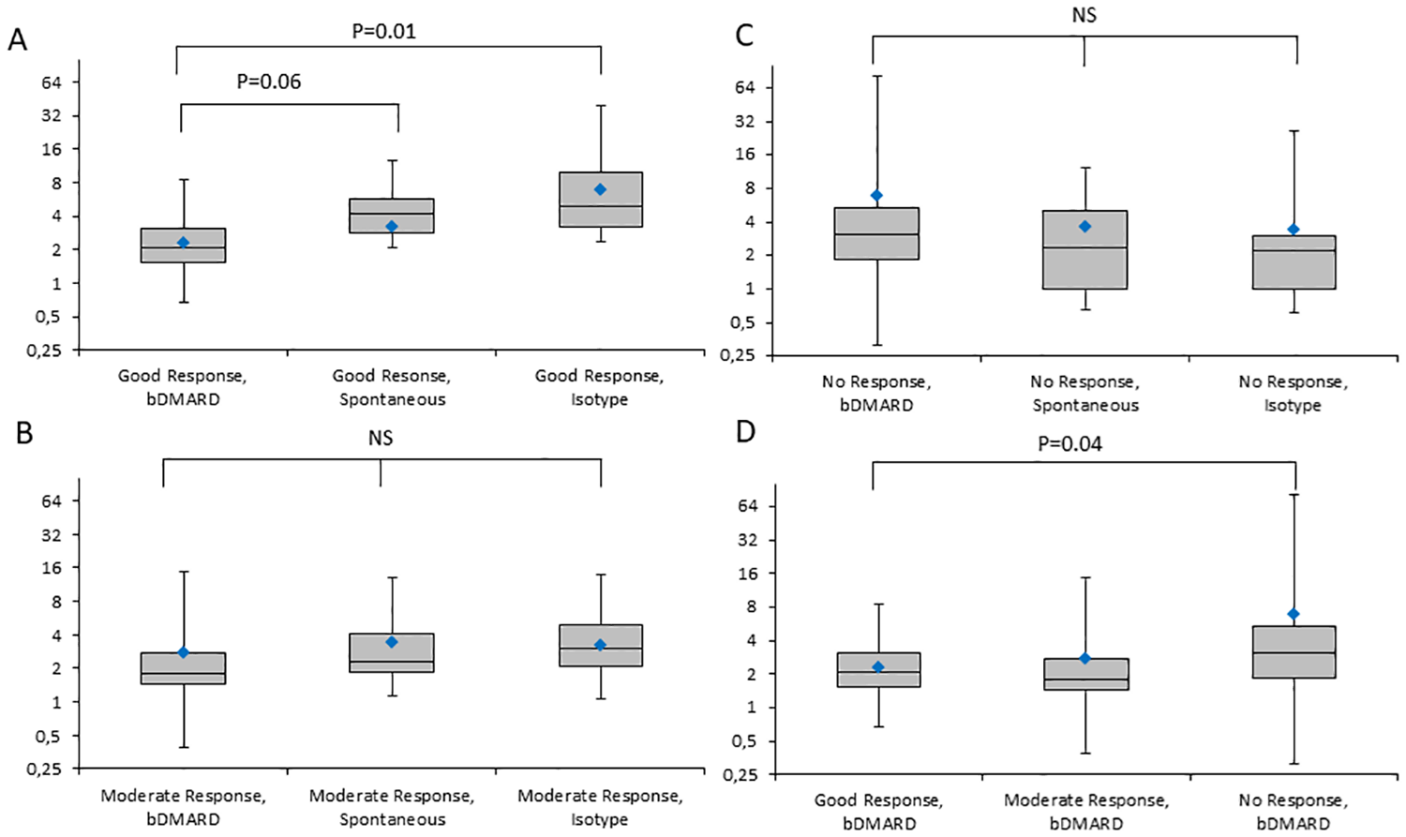
Box plots illustrating changes in fold changes in explant production of IL-6 with regards to in vitro interventions and EULAR responses. Box plots illustrating changes in fold changes in explant production of IL-6 with regards to *in vitro* interventions and EULAR responses. ‘Diamond’ corresponds to mean fold change of IL-6. Only statistically significant P-values are depicted. a) bDMARD treated samples, b) spontaneous release, c) isotype control, d) good responders, e) moderate responders, f) none responders. bDMARD = *in vitro* added biologic disease modifying anti-rheumatic drug; IL-6 = interleukin 6; pg = picogram; Isotype = isotype matched IgG. NS = statistically non-significant, P>0.05.

Correlation coefficients for spontaneous and bDMARD-treated explants with regards to IL-6 and changes in DAS-28 were significant (P = 0.03 for both), with ρ = 0.56 and ρ = 0.57. Scatterplots of changes in IL-6 from bDMARD-treated samples and changes in DAS-28, CF_max_ and BME are given in Fig 3. Changes in MCP-1 production were not correlated to changes in DAS-28 (ρ = 0.37, P = 0.15).

### Imaging and explant data

Of the 40 cultures, 38 explant cultures (15 patients) had matching CDUS data at baseline and follow-up (two cultures from one patient had no CDUS data at follow-up due to logistics). Baseline CDUS was median CF_max_ of 9% (IQR 0% to 16%) with ranges from no Doppler activity (10 explants) to high activity (3 explants). At follow-up, Doppler activity had decreased in the areas corresponding to approximately half of the explants (18/38). All10 sites with absence of Doppler activity at baseline remained Doppler negative at follow up. An increase in CF_max_ was seen at the remaining 10 sites.

A total of 28 cultures (from 11 patients) had MRI data available at both baseline and follow-up. The causes for missing MRI data were logistics at the Department of Radiology in three cases, one incident of claustrophobia, and one patient with a contra-indication to MRI (coronary stent).

24 culture positions showed moderate to severe synovitis. Severe synovitis was the predominant finding accounting for 61% (17/28) of the explant material. Three cultures from the same patient had no MRI signs of synovitis.

The presence of BME was moderate at baseline with a median score of 1.75 (IQR: 1.0 to 2.75), and all except 2 patients (3 explant cultures)) had BME present at baseline. At follow-up, the changes were polarized with 50% of joint positions having experienced a decrease in BME, whereas other 40% showed an unchanged pathological score or an increase in BME score. On a group basis, a median decrease of -0.25 point (IQR: -1.75 to 0.0) in BME was observed.

All but one patient exhibited erosions at baseline. The extent of erosions was moderate with a median of 1.75 on the RAMRIS score (IQR: 1.0 to 2.5). A slight increase in eroded bone developed during the study period with a median RAMRIS erosion score of 2.0 eroded bone at follow up (IQR: 1.3 to 2.7). 20 of the 28 explant cultures came from joint positions showing an increase in erosion, whereas only one position showed a decrease in RAMRIS erosion score at follow up. All changes were moderate with changes less than 5 percent, apart from two explant cultures with an increase of 8 and 10% in the corresponding anatomical region, respectively. A detailed overview of the imaging data is found in Table 2.

**Table 2:** An overview of global imaging activity during the study period.

Variables		*Baseline*					*Follow up*	*Change*
	Observations [No. Patients]	Median [Q_1_;Q_3_] (min; max)		Observations [No. Patients]			Median [Q_1_;Q_3_] (min; max)	Median [Q_1_;Q_3_] (min; max)
Imaging								
CF_max_ (0–1)								
Wrist	37[14]	0.09 [0.02; 0.22] (0.0; 0.54)		35 [13]			0.0 [0.0; 0.17] (0.0; 0.54)	0.0 [-0.15; 0.003] (-0.44; 0.22)
MCP	3[2]	0.08 [0.0; 0.52] (0.0; 0.52)		3 [2]			0.14 [0;0.16] (0.0; 0.16)	0.0 [-0.35; 0.06] (-0.35; 0.06))
Total	40[16]	0.09 [0.01; 0.22] (0.0; 0.54)		38[15]			0.0 [0.0; 0.16] (0.0; 0.54)	0.0 [-0.15; 0.003] (-0.44; 0.22)
RAMRIS synovitis score (0–3)								
Wrist	26[10]	3.0 [2.0; 3.0] (0.0; 3.0)		26[10]			[1.0; 2.0] (1.0; 3.0)	-1.0 [-1.0; 2.0] (-2.0; 1.0)
MCP	2[1]	2.5 [2.0; 3.0] (2.0; 3.0)		2[1]			2.5 [2.0; 3.0] (2.0; 3.0)	0.0 [0.0; 0.0] (0.0;0.0)
Total	28[11]	3.0 [2.0; 3.0] (0.0; 3.0)		28[11]			1.5 [1.0; 2.0] (1.0;3.0)	-1.0[-1.0; 0.0] (-2.0; 1.0)
RAMRIS BME score (0–3)								
Wrist	26[10]	1.75 [1.0; 2.5] (0.0; 3.0)		26[10]	0.75 [0.0; 2.0] (1.0; 3.0)			-0.5 [-2.0;0.0] (-3.0; 0.5)
MCP	2[1]	1.5 [0.0; 3.0] (0.0; 3.0)		2[1]	2.3[1.5; 3.0] (1.5; 3.0)			0.75 [0.0; 1.5] (0;1.5)
Total	28[11]	1.8 [1.0; 2.8] (0.0; 3.0)		28[11]	0.9 [0.0; 1.5] (0.0; 3.0)			-0.3 [-1.8; 0.0] (-3.0;1.5)
RAMRIS erosion score (0–3)								
Wrist	26[10]	1.75 [1.0; 2.5] (0.0; 3.5)		26[10]	2.0 [1.25;2.5] (0.0; 3.5)			0.0 [0.0; 0.5] (-0.5; 1.0)
MCP	2[1]	2.0[1.5; 2.5] (1.5; 2.5)		2[1]	2.3 [1.5;3.0] (1.5; 3.0)			0.3 [0.0; 0.5] (0.0; 0.5)
Total	28[11]	1.8 [1.0;2.5] (0;3.5)		28[11]	2.0 [1.3; 2.7] (0.0;3.5)			0.0 [0;0.50] (-0.5;1.0)

Overview of imaging activity among the 16 RA patients who fulfilled the inclusion criteria and were initiated in biological treatment. Data presented as median [IQR] and (min; max). CFmax = colour fraction measured in the systole; IQR = Interquartile range (1st quartile; 3rd quartile); Max. = maximum; MCP = Metacarpophalangeal joint; Min. = minimum; no. = number PIP = Proximal interphalangeal joint; RAMRIS BME Score = Rheumatoid Arthritis Magnetic Resonance Bone Marrow Oedema Score.

#### Correlations of mediator production of explants with change in doppler activity

Spearman correlations showed statistically significant associations for fold change in spontaneous IL-6 release (ρ = 0.68, P = 0.04) and borderline significant for bDMARD-treated explants (ρ = 0.53, P = 0.06; P = 0.04 in the non-reduced model). Isotype control was not statistically significantly associated (ρ = 0.03, P = 0.24).

Change in MCP-1 production was not significantly associated with changes in CF_max_ for any of the interventions: bDMARD-treated explants (ρ = 0.34, P = 0.17), spontaneous release (ρ = 0.36, P = 0.71) and isotype controls (ρ = -0.03, P = 0.89). S3–S6 Tables offer details on the mixed model covariate elimination steps.

### Correlations of explant mediator production with MRI

The strongest signals came from changes in bDMARD-treated explants for increase in IL-6 and MCP-1, which showed a moderate degree of correlation of (ρ = 0.56 P = 0.03) and (ρ = 0.49 P = 0.01) for changes in RAMRIS BME, respectively. The correlations were insignificant between RAMRIS and the explants’ spontaneous release, as well as isotype controls’, for IL-6 and MCP-1. S3–S6 Tables offer details on the mixed model covariate elimination steps.

Mixed model analyses could not be performed for RAMRIS synovitis score or RAMRIS erosion score due to failure of the model control criteria.

Correlations were weak to moderate between changes in the RAMRIS synovitis score and changes in bDMARD-treated explants’ production of IL-6 or MCP-1. The highest correlation coefficient was seen for bDMARD-treated samples for changes in IL-6 (ρ = 0.30) and spontaneous release of MCP-1 (ρ = 0.17). When explant data from the different joint positions was averaged per patient, all P-values for the correlations using Spearman’s rank test were insignificant for both changes in RAMRIS BME and erosion scores (data not shown).

The changes in RAMRIS erosion score did not correlate significantly with any changes in cytokine production.

## Discussion

In this study, explants cultured from RA joints obtained prior to bDMARD *in vivo* therapy demonstrated that change in IL-6 production significantly corresponded to both the overall clinical and the imaging effect determined following a median of 7 months of treatment. Thus, explants from non-responders based on the EULAR response criteria had a significantly higher IL-6 production in bDMARD-treated samples than samples from EULAR good responders. Furthermore, IL-6 production was significantly lower in bDMARD-treated samples from good responders than in matched isotype controls. In contrast, IL-6 production was not suppressed in samples from moderate responders and non-responders, indicating that the explant model provided disease-relevant information. No significant associations were found between EULAR response or DAS-28 changes and bDMARD effects on explant MCP-1 production.

In recent years, multi-biomarker tests based on blood tests have shown good correlations with clinical disease activity measures. Furthermore, IL-6 was shown to correlate with DAS-28, TJC and SJC[9,21,22]. Plasma IL-6 levels have been associated with clinical remission in an Infliximab-treated RA cohort which underlines the qualities of IL-6 as a biomarker of disease activity and treatment response [23]. Baseline CRP and DAS-28 did not reveal statistically significantly differences according to EULAR response. However, baseline DAS-28 was borderline significantly lower among non-responders compared to good responders (P = 0.07, non-parametric testing) indicating that clinical evaluation is still an important prognostic feature.

Explant production of both IL-6 and MCP-1 correlated with imaging responses at the explant sites following a median of 7 months of bDMARD treatment. This indicates that whole tissue synovial explants may provide valuable information concerning the subsequent bDMARD effect *in vivo*, both at the local joint and for the overall disease activity.

The explants’ mediator release correlated with changes in CDUS and bone marrow oedema in contrast to MRI-detected synovitis and erosive changes. The RAMRIS synovitis score evaluates synovial volume. The score is thus likely to have a higher degree of bias introduced by the synovectomy, since the synovial volume becomes reduced by this procedure. In contrast, CDUS activity was calculated as the systolic pixel/gray scale fraction (CF_max_), and this may not be influenced to the same extent by a decrease in synovial volume. With respect to erosions, these changes are less than the other imaging parameters and lack of significant associations may have been caused by the small sample size and relatively short follow period [24–27].

Overall, the synovectomy procedure on a single small joint only introduced a limited bias on DAS-28, which was included on average 7 swollen and painful joints at the time of synovectomy. With respect to the Doppler findings of the target joint, changes may have been introduced by the surgical procedure both causing excess flow from reparative changes in the tissue as well as the opposite, i.e. reduced flow due to removal of affected synovium.

The imaging data appeared representative for a general RA cohort, since both baseline values and changes in imaging correlations to clinical disease-activity outcome measures corresponded to previous observations in RA cohorts[28,29].

### Limitations

The vast majority of samples were obtained from wrists where the synovectomy material came from shavings at the dorsal side. Thus, it cannot be ruled out that synovium from neighbouring sites may have contributed to imaging pathology. Due to logistic limitations it was not possible to uniform the timing of follow up visits, wherefore there was quite a big difference in length of bDMARD therapies among patients. The impact of the different intervals was limited by the fact that no patients were switched to other bDMARD therapy between baseline and follow up visits.

This pilot study was not designed to identify the optimal in vitro dose of bDMARD and isotype control. The choice of an IgG light chain isotype would not have detected a possible unspecific *in vitro* Fc mediated effect of the bDMARDs consisting of whole antibodies. This is however not likely to be the case since the bDMARDs are highly specific for their molecular targets.

Other studies have shown that a dose-dependent suppression of explant culture mediators can be observed using increasing doses of anti-TNFα inhibitors as high as 100μg/ml[30,31]. The choice of a minimum use of 10 μg/ml of bDMARD here seemed appropriate according to previous studies, where concentration varied between 1 μg/ml and 10μg/ml[12,30–34]. Among patients with data from samples treated with both 10 μg/ml and 50 μg/ml we did not see a clear indication of superior suppression of the higher bDMARD doses. Our *in vitro* design did not account for *in vivo* differences in dosage of the various bDMARDs. This could pose a potential bias when translating from *in vitro* to *in vivo* response.

Further studies are now needed to identify the optimal *in vitro* doses of the different bDMARDs.

Although the surgeon had full visible overview during the arthroscopic procedure and was guided by the ultrasound description, an accurate anatomical match between the site of synovectomy and the imaging data was not possible.

Another study limitation was the heterogeneity regarding previous treatment with bDMARDs. Approximately two thirds of the patients were bDMARD naïve and one third bDMARD failures who were opted for treatment switching. However, synovectomy in the latter group was only performed after a pause in treatment with no trace expected of the former bDMARD. We therefore believe that a difference in treatment response biased by previous bDMARD exposure is limited.

The small sample number increased the risk of type II errors. Thus, the model could only detect a statistical difference between EULAR none responders and EULAR good responders, but not differentiate between all three EULAR response types.

Due to financial limitations, it was unfortunately not possible to analyse more mediators.

### Strengths

The patients recruited for the study had active systemic disease and were scheduled for bDMARD treatment. This is in contrast to most previous studies of biopsies that were mainly obtained from end-stage disease, which may not be representative for the general RA inflammation. The use of hand joints reduced a risk of bias from concomitant osteoarthritis that might otherwise blur RA specific inflammatory signals. Explants based on synovectomy products from site-specific areas in small joints by needle arthroscopy ensured an optimal harvest of all relevant synovium. The arthroscopy procedure enabled full visual overview during the synovectomy and thereby optimal conditions for mapping the synovectomized sites with the corresponding anatomical areas on imaging. Finally, the use of intact tissue cultured on bone without addition of exogenous immuno-stimulation and enzyme digestion does mimic the *in vivo* situation as far as it is possible[35–38].

## Conclusions

To our knowledge this is the first study investigating the association of short term change in synovial mediator production *in vitro* with long term clinical outcomes in RA patients treated with bDMARDs.

The model suggests that short term changes in the synovium are associated with clinical outcome following treatment. The results are encouraging concerning use of explant models in the ongoing process of clarifying the underlying pathology in RA and identification of future biomarkers that could pave the road for patient-tailored treatment options.

## Supporting information

S1 Table ‘Overview of the anatomical landmarks used for mapping the anatomical origin of the explants with the corresponding anatomical location on imaging.’ Overview of the anatomical landmarks used for mapping the anatomical origin of the explants with the corresponding anatomical location on imaging.
(DOCX)

S2 Table ‘Overview of IL-6 and MCP-1 explant changes according to the type of in vitro intervention and EULAR response to bDMARD treatment.’ Changes in explant fold change of IL-6 and MCP-1 during the 2 week culture period grouped by EULAR DAS-28 response criteria. bDMARD = explants treated with 10μg/ml biologic DMARD; DMARD = disease modifying anti-rheumatic drug; IL-6 = Interleukin 6; Isotype = Matched isotype control, 10μg/ml; Max = maximum value; MCP-1 = macrophage chemoattractant protein 1; Min = minimum value; Ne = Number of explants; Nmis = number missing explants due to baseline value < 20% of overall average; NP = number of patients; Response = EULAR DAS-28 response criteria; Spontaneous = fold change in untreated explant IL-6 production, STDV = standard deviation.
(DOCX)

S3 Table Fold change in RA explant IL-6 release vs. change in CFmax upon biologic DMARD treatment. Stepwise covariate elimination. This table depicts the statistical associations between CDUS (ΔCFmax) activity and synovial explant mediator fold change (2 weeks culture concentration divided by the concentration at 72h of culture) for the spontaneous release of mediators, mediator release of cultures with bio.DMARD (10μg/ml) and isotype control (10μg/ml). A mixed model has been used for the statistical analysis, P<0.05 was considered significant. In the reduced model covariates were excluded if P>0.10. All of the four pre-specified covariates, tested in the models, are illustrated above. RAMRIS = Rheumatoid arthritis magnetic resonance score; syno = synovitis; Log10 = 10 logarithm; √ = square root; Inv = Inverted.Covariates included in the statistical model: Joint Synovectomized = Wrist, MCP or PIP; Synovectomy position = Ulnar, central, radial or mixed for pooled synovectomy positions; Side = left or right; bDMARD = biologic disease modifying anti-rheumatic drugs; CFmax = maximal color fraction; Δ = change in imaging variable after a minimum of three months treatment with a biologic DMARD; IL-6 = Interleukin 6; MCP = metacarpophalangeal joint,; PIP = Proximal interphalangeal joint.
(DOC)

S4 Table Fold change in RA explant IL-6 release vs. change in RAMRIS BME score upon biologic DMARD treatment. Stepwise covariate elimination. This table depicts the statistical associations between RAMRIS BME score and synovial explant mediator fold change (2 weeks culture concentration divided by the concentration at 72h of culture) for the spontaneous release of mediators, mediator release of cultures with bDMARD (10μg/ml) and isotype control (10μg/ml). A mixed model has been used for the statistical analysis, P<0.05 was considered significant. In the reduced model covariates were excluded if P>0.10. All of the four pre-specified covariates, tested in the models, are illustrated above. RAMRIS = Rheumatoid arthritis magnetic resonance score; BME = Rheumatoid arthritis magnetic resonance score bone marrow edema score; Log10 = 10 logarithm; √ = square root; Inv = Inverted. Covariates included in the statistical model: Joint Synovectomized = Wrist, MCP or PIP; Synovectomy position = Ulnar, central, radial or mixed for pooled synovectomy positions; Side = left or right; bDMARD = biologic disease modifying anti-rheumatic drugs; IL-6 = Interleukin 6, MCP = metacarpophalangeal joint,; PIP = Proximal interphalangeal joint.
(DOCX)

S5 Table ‘Fold change in RA explant MCP-1 release vs. change in CFmax upon biologic DMARD treatment. Stepwise covariate elimination.’ This table depicts the statistical associations between CDUS (ΔCFmax) activity and synovial explant mediator fold change (2 weeks culture concentration divided by the concentration at 72h of culture) for the spontaneous release of mediators, mediator release of cultures with bDMARD (10μg/ml) and isotype control (10μg/ml). A mixed model has been used for the statistical analysis, P<0.05 was considered significant. In the reduced model covariates were excluded if P>0.10. All of the four pre-specified covariates, tested in the models, are illustrated above. Inv = Inverted; Log10 = 10 logarithm; √ = square root; syno = synovitis.Covariates included in the statistical model: Joint Synovectomized = Wrist, MCP or PIP; Synovectomy position = Ulnar, central, radial or mixed for pooled synovectomy positions; Side = left or right; bDMARD = biologic disease modifying anti-rheumatic drugs; CFmax = maximal color fraction; Δ = change in imaging variable after a minimum of three months treatment with a bDMARD; MCP-1 = monocyte chemoatrractant protein 1; MCP = metacarpophalangeal joint,; PIP = Proximal interphalangeal joint.
(DOC)

S6 Table ‘Fold change in RA explant MCP-1 release vs. change in RAMRIS BME score upon biologic DMARD treatment. Stepwise covariate elimination.’ This table depicts the statistical associations between the change in RAMRIS BME score in bDMARD treated RA patients (N = 11, 28 explants) and change in synovial explant mediator release after 2 weeks of culture. A mixed model has been used for the statistical analysis, P<0.05 was considered significant. In the reduced model covariates were excluded if P>0.10. All of the four pre-specified covariates, tested in the models, are illustrated above. bDMARD = biologic disease modifying anti-rheumatic drugs; RAMRIS BME = Rheumatoid Arthritis Magnetic Resonance Imaging Score for Bone Marrow Oedema. Log10 = 10 logarithm, √ = square root. Inv = Inverted. * = model control failed normal distribution of residuals. Covariates included in the statistical model: Joint Synovectomized = Wrist, MCP or PIP; Synovectomy position = Ulnar, central, radial or mixed for pooled synovectomy positions; Side = left or right; MCP-1 = Monocyte Chemoattractant Protein 1; MCP = metacarpophalangeal joint,; PIP = Proximal interphalangeal joint.
(DOCX)
