# Supplementary material for: Association between IL-6 production in synovial explants from rheumatoid arthritis patients and clinical and imaging response to biologic treatment: A pilot study
Source: PLoS One. 2018 May 22;13(5):e0197001. doi: 10.1371/journal.pone.0197001 (PMC5963776; doi:10.1371/journal.pone.0197001)
Supplement: S1 Table — Overview of the anatomical landmarks used for mapping the anatomical origin of the explants with the corresponding anatomical location on imaging. (DOCX) [file pone.0197001.s001.docx]

Supporting Information S1. Overview of the anatomical landmarks used for mapping the anatomical origin of the explants with the corresponding anatomical location on imaging.

| **Position** | **Radiocarpal level** | **Mid-carpal level** |
| --- | --- | --- |
| Radial | Distal radius and scaphoid bone (RCR) | Scaphoid and trapezoid bones (MCR) |
| Central | Distal radius and lunate bone (RCC) | Capitate and lunate bones (MCC) |
| Ulnar | Ulnar meniscus and triquetate bone (RCU) | Triquetate and hamatate bones (MCU) |
| Supporting Information S1. Overview of the anatomical landmarks used for mapping the explant material with the corresponding anatomy on imaging. |  |  |
