# Supplementary material for: Association between IL-6 production in synovial explants from rheumatoid arthritis patients and clinical and imaging response to biologic treatment: A pilot study
Source: PLoS One. 2018 May 22;13(5):e0197001. doi: 10.1371/journal.pone.0197001 (PMC5963776; doi:10.1371/journal.pone.0197001)
Supplement: S2 Table — Changes in explant fold change of IL-6 and MCP-1 during the 2 week culture period grouped by EULAR DAS-28 response criteria. bDMARD = explants treated with 10μg/ml biologic DMARD; DMARD = disease modifying anti-rheumatic drug; IL-6 = Interleukin 6; Isotype = Matched isotype control, 10μg/ml; Max = maximum value; MCP-1 = macrophage chemoattractant protein 1; Min = minimum value; Ne = Number of explants; Nmis = number missing explants due to baseline value < 20% of overall average; NP = number of patients; Response = EULAR DAS-28 response criteria; Spontaneous = fold change in untreated explant IL-6 production, STDV = standard deviation. (DOCX) [file pone.0197001.s002.docx]

| **Variable** | **Response** | N_e_ (N_p_) | N_mis_ | **Median** | **Mean** | **STDV** | **Min** | **Max** |
| --- | --- | --- | --- | --- | --- | --- | --- | --- |
| IL-6 Spontaneous | None | 41(6) | 19 | 2.32 | 3.62 | 3.09 | 0.65 | 12.26 |
| IL-6 Bio | None | 30(6) | 30 | 3.09 | 6.92 | 14.61 | 0.72 | 80.83 |
| IL-6 Isotype | None | 23(6) | 37 | 2.21 | 3.37 | 5.33 | 0.62 | 26.16 |
|  |  |  |  |  |  |  |  |  |
| IL-6 Spontaneous | Moderate | 21(4) | 19 | 1.86 | 3.38 | 3.23 | 0.76 | 12.76 |
| IL-6 Bio | Moderate | 19(4) | 21 | 1.46 | 2.73 | 3.33 | 0.08 | 14.43 |
| IL-6 Isotype | Moderate | 27(4) | 13 | 2.08 | 3.23 | 3.31 | 0.09 | 12.71 |
|  |  |  |  |  |  |  |  |  |
| IL-6 Spontaneous | Good | 28(6) | 32 | 2.85 | 3.24 | 2.27 | 0.68 | 11.23 |
| IL-6 Bio | Good | 30(6) | 30 | 1.55 | 2.27 | 2.02 | 0.12 | 8.03 |
| IL-6 Isotype | Good | 18(6) | 42 | 3.17 | 6.78 | 9.26 | 0.63 | 37.99 |
|  |  |  |  |  |  |  |  |  |
| MCP-1 Spontaneous | None | 46(6) | 14 | 4.94 | 8.38 | 15.74 | 0.70 | 106.4 |
| MCP-1 Bio | None | 42(6) | 18 | 5.35 | 7.99 | 12.25 | 1.00 | 75.56 |
| MCP-1 Isotype | None | 32(6) | 28 | 4.08 | 6.19 | 7.85 | 1.00 | 40.76 |
|  |  |  |  |  |  |  |  |  |
| MCP-1 Spontaneous | Moderate | 21(4) | 19 | 5.84 | 6.23 | 2.65 | 2.10 | 11.81 |
| MCP-1 Bio | Moderate | 25(4) | 15 | 4.57 | 6.68 | 6.35 | 0.21 | 29.00 |
| MCP-1 Isotype | Moderate | 32(4) | 8 | 5.63 | 6.47 | 5.55 | 0.35 | 26.37 |
|  |  |  |  |  |  |  |  |  |
| MCP-1 Spontaneous | Good | 36(6) | 24 | 3.95 | 5.33 | 4.63 | 1.00 | 22.37 |
| MCP-1 Bio | Good | 41(6) | 19 | 2.71 | 6.12 | 8.64 | 1.00 | 46.85 |
| MCP-1 Isotype | Good | 31(6) | 29 | 5.72 | 7.09 | 6.33 | 0.42 | 27.30 |

S2 Table. Overview of IL-6 and MCP-1 explant changes according to the type of in vitro intervention and EULAR response to bDMARD treatment.

S2 Table. Changes in explant fold change of IL-6 and MCP-1 during the 2 week culture period grouped by EULAR DAS-28 response criteria.

bDMARD= explants treated with 10µg/ml biologic DMARD; DMARD = disease modifying anti-rheumatic drug; IL-6 = Interleukin 6; Isotype = Matched isotype control, 10µg/ml; Max = maximum value; MCP-1 = macrophage chemoattractant protein 1 ; Min = minimum value; Ne= Number of explants; Nmis = number missing explants due to baseline value < 20% of overall average; NP = number of patients; Response = EULAR DAS-28 response criteria; Spontaneous = fold change in untreated explant IL-6 production, STDV = standard deviation.
