# Supplementary material for: Association between IL-6 production in synovial explants from rheumatoid arthritis patients and clinical and imaging response to biologic treatment: A pilot study
Source: PLoS One. 2018 May 22;13(5):e0197001. doi: 10.1371/journal.pone.0197001 (PMC5963776; doi:10.1371/journal.pone.0197001)
Supplement: S3 Table — This table depicts the statistical associations between CDUS (ΔCFmax) activity and synovial explant mediator fold change (2 weeks culture concentration divided by the concentration at 72h of culture) for the spontaneous release of mediators, mediator release of cultures with bio.DMARD (10μg/ml) and isotype control (10μg/ml). A mixed model has been used for the statistical analysis, P<0.05 was considered significant. In the reduced model covariates were excluded if P>0.10. All of the four pre-specified covariates, tested in the models, are illustrated above. RAMRIS = Rheumatoid arthritis magnetic resonance score; syno = synovitis; Log10 = 10 logarithm; √ = square root; Inv = Inverted.Covariates included in the statistical model: Joint Synovectomized = Wrist, MCP or PIP; Synovectomy position = Ulnar, central, radial or mixed for pooled synovectomy positions; Side = left or right; bDMARD = biologic disease modifying anti-rheumatic drugs; CFmax = maximal color fraction; Δ = change in imaging variable after a minimum of three months treatment with a biologic DMARD; IL-6 = Interleukin 6; MCP = metacarpophalangeal joint,; PIP = Proximal interphalangeal joint. (DOC) [file pone.0197001.s003.doc]

**Additional File 3a. Fold change in RA explant IL-6 release vs. Change in CF_max_ upon biologic DMARD treatment. Stepwise covariate elimination**

| **Dependent variable** | **Full model**  **(P-value)** | **1^st^ Reduced model (P-value )** | **2nd Reduced model (P-value )** | **3rd Reduced model (P-value )** |
| --- | --- | --- | --- | --- |
| **IL-6_spontaneous_** | Joint Synovectomized  (P=0.46) |  |  |  |
| (Approx. Spearman:  Rho=0.68)  N= 15, obs. =38 | Synovectomy position  (P=0.14) | Synovectomy position  (P=0.33) | Synovectomy position  (P=0.34) |  |
|  | Side  (P=0.25) | Side  (P=0.69) |  |  |
|  | **Δ**CFmax  (P=0.06) | **Δ**CFmax  (P=0.05) | **Δ**CFmax  (P=0.04) | **Δ**CFmax  (P=0.04) |
| **Inv_**√**(IL-6_bio.dmard_)** | Joint Synovectomized  (P=0.97) |  |  |  |
| (Approx. Spearman:  Rho=0.53)  N= 15, obs. =36 | Synovectomy position  (P=0.43) | Synovectomy position  (P=0.37) | Synovectomy position  (P=0.37) |  |
|  | Side  (P=0.69) | Side  (P=0.66) |  |  |
|  | **Δ**CFmax  (P=0.04) | **Δ**CFmax  (P=0.03) | **Δ**CFmax  (P=0.04) | **Δ**CFmax  (P=0.06) |
| √(IL-6**_Isotype control_)** | Joint Synovectomized  (P=0.68 |  |  |  |
| (Approx. Spearman:  Rho=0.03)  N= 15, obs. =38 | Synovectomy position  (P=0.52 ) | Synovectomy position  (P=0.53 ) | Synovectomy position  (P=0.33) |  |
|  | Side  (P=0.55) | Side  (P=0.61) |  |  |
|  | **Δ**CFmax  (P=0.46 ) | **Δ**CFmax  (P=0.36 ) | **Δ**CFmax  (P=0.23 ) | **Δ**CFmax  (P=0.24 ) |
|  |  |  |  |  |

This table depicts the statistical associations between CDUS (**Δ**CFmax) activity and synovial explant mediator fold change (2 weeks culture concentration divided by the concentration at 72h of culture) for the spontaneous release of mediators, mediator release of cultures with bio.DMARD (10µg/ml) and isotype control (10µg/ml). A mixed model has been used for the statistical analysis, P<0.05 was considered significant. In the reduced model covariates were excluded if P>0.10. All of the four pre-specified covariates, tested in the models, are illustrated above.

RAMRIS = Rheumatoid arthritis magnetic resonance score; syno = synovitis; Log_10_= 10 logarithm; **√ =** square root; Inv = Inverted.

Covariates included in the statistical model: Joint Synovectomized = Wrist, MCP or PIP; Synovectomy position = Ulnar, central, radial or mixed for pooled synovectomy positions; Side = left or right; bio. DMARD = biologic disease modifying anti-rheumatic drugs; CFmax= maximal color fraction; Δ= change in imaging variable after a minimum of three months treatment with a biologic DMARD; IL-6 = Interleukin 6; MCP = metacarpophalangeal joint; PIP = Proximal interphalangeal joint.
