# Supplementary material for: Association between IL-6 production in synovial explants from rheumatoid arthritis patients and clinical and imaging response to biologic treatment: A pilot study
Source: PLoS One. 2018 May 22;13(5):e0197001. doi: 10.1371/journal.pone.0197001 (PMC5963776; doi:10.1371/journal.pone.0197001)
Supplement: S4 Table — This table depicts the statistical associations between RAMRIS BME score and synovial explant mediator fold change (2 weeks culture concentration divided by the concentration at 72h of culture) for the spontaneous release of mediators, mediator release of cultures with bDMARD (10μg/ml) and isotype control (10μg/ml). A mixed model has been used for the statistical analysis, P<0.05 was considered significant. In the reduced model covariates were excluded if P>0.10. All of the four pre-specified covariates, tested in the models, are illustrated above. RAMRIS = Rheumatoid arthritis magnetic resonance score; BME = Rheumatoid arthritis magnetic resonance score bone marrow edema score; Log10 = 10 logarithm; √ = square root; Inv = Inverted. Covariates included in the statistical model: Joint Synovectomized = Wrist, MCP or PIP; Synovectomy position = Ulnar, central, radial or mixed for pooled synovectomy positions; Side = left or right; bDMARD = biologic disease modifying anti-rheumatic drugs; IL-6 = Interleukin 6, MCP = metacarpophalangeal joint,; PIP = Proximal interphalangeal joint. (DOCX) [file pone.0197001.s004.docx]

**S4 Table. Fold change in RA explant IL-6 release vs. Change in RAMRIS BME score upon biologic DMARD treatment. Stepwise covariate elimination**

| **Dependent variable** | **Full model**  **(P-value)** | **1^st^ Reduced model (P-value )** | **2nd Reduced model (P-value )** | **3rd Reduced model (P-value )** |
| --- | --- | --- | --- | --- |
| **Log10(IL-6_spontaneous_)** | Joint Synovectomized  (P=0.32) | Joint Synovectomized  (P=0.28) | Joint Synovectomized  (P=0.36) |  |
| (Approx. Spearman:  Rho=0.37)  N= 9, obs. = 26 | Synovectomy position  (P=0.81) |  |  |  |
|  | Side  (P=0.42) | Side  (P=0.47) |  |  |
|  | **Δ**BME  (P=0.18) | **Δ**BME  (P=0.09) | **Δ**BME  (P=0.09) | **Δ**BME  (P=0.10) |
| **IL-6_bio.dmard_** | Joint Synovectomized  (P=0.46) | Joint Synovectomized  (P=0.43) | Joint Synovectomized  (P=0.27) |  |
| (Approx. Spearman:  Rho=0.56)  N= 11, obs. =28 | Synovectomy position  (P=0.65) | Synovectomy position  (P=0.65) |  |  |
|  | Side  (P=0.97) |  |  |  |
|  | **Δ**BME  (P=0.07) | **Δ**BME  (P=0.06) | **Δ**BME  (P=0.08) | **Δ**BME  (P=0.03) |
| Log10(IL-6**_Isotype control_)** | Joint Synovectomized  (P=0.32) | Joint Synovectomized  (P=0.30) | Joint Synovectomized  (P=0.14) |  |
| (Approx. Spearman:  Rho=0.48)  N= 11, obs. =28 | Synovectomy position  (P=0.63 ) | Synovectomy position  (P=0.61 ) |  |  |
|  | Side  (P=0.97) |  |  |  |
|  | **Δ**BME  (P=0.13 ) | **Δ**BME  (P=0.12 ) | **Δ**BME  (P=0.21 ) |  |
|  |  |  |  |  |

This table depicts the statistical associations between RAMRIS BME score and synovial explant mediator fold change (2 weeks culture concentration divided by the concentration at 72h of culture) for the spontaneous release of mediators, mediator release of cultures with bDMARD (10µg/ml) and isotype control (10µg/ml). A mixed model has been used for the statistical analysis, P<0.05 was considered significant. In the reduced model covariates were excluded if P>0.10. All of the four pre-specified covariates, tested in the models, are illustrated above.

RAMRIS = Rheumatoid arthritis magnetic resonance score; BME = Rheumatoid arthritis magnetic resonance score bone marrow edema score; Log_10_= 10 logarithm; **√ =** square root; Inv = Inverted.

Covariates included in the statistical model: Joint Synovectomized = Wrist, MCP or PIP; Synovectomy position = Ulnar, central, radial or mixed for pooled synovectomy positions; Side = left or right; bDMARD = biologic disease modifying anti-rheumatic drugs; IL-6 = Interleukin 6; MCP = metacarpophalangeal joint; PIP = Proximal interphalangeal joint.
