# Supplementary material for: Association between IL-6 production in synovial explants from rheumatoid arthritis patients and clinical and imaging response to biologic treatment: A pilot study
Source: PLoS One. 2018 May 22;13(5):e0197001. doi: 10.1371/journal.pone.0197001 (PMC5963776; doi:10.1371/journal.pone.0197001)
Supplement: S6 Table — This table depicts the statistical associations between the change in RAMRIS BME score in bDMARD treated RA patients (N = 11, 28 explants) and change in synovial explant mediator release after 2 weeks of culture. A mixed model has been used for the statistical analysis, P<0.05 was considered significant. In the reduced model covariates were excluded if P>0.10. All of the four pre-specified covariates, tested in the models, are illustrated above. bDMARD = biologic disease modifying anti-rheumatic drugs; RAMRIS BME = Rheumatoid Arthritis Magnetic Resonance Imaging Score for Bone Marrow Oedema. Log10 = 10 logarithm, √ = square root. Inv = Inverted. * = model control failed normal distribution of residuals. Covariates included in the statistical model: Joint Synovectomized = Wrist, MCP or PIP; Synovectomy position = Ulnar, central, radial or mixed for pooled synovectomy positions; Side = left or right; MCP-1 = Monocyte Chemoattractant Protein 1; MCP = metacarpophalangeal joint,; PIP = Proximal interphalangeal joint. (DOCX) [file pone.0197001.s006.docx]

**S6 Table. Fold change in RA explant MCP-1 release vs. Change in RAMRIS BME score upon bDMARD treatment. Stepwise covariate elimination**

| **Dependent variable** | **Full model**  **(P-value)** | **1^st^ Reduced model (P-value )** | **2nd Reduced model (P-value )** | **3rd Reduced model (P-value )** |
| --- | --- | --- | --- | --- |
| **Inv_log10(MCP-1_spontaneous_)** | Joint Synovectomized  (P=0.19) | Joint Synovectomized  (P=0.17) | Joint Synovectomized  (P=0.13) |  |
| **(Approx. Spearman:**  **Rho=0.37)**  **N= 9, obs. = 26** | Synovectomy position  (P=0.48) | Synovectomy position  (P=0.48) |  |  |
|  | Side  (P=0.92) |  |  |  |
|  | **Δ**BME  (P=0.12) | **Δ**BME  (P=0.12) | **Δ**BME  (P=0.21) |  |
| **MCP-1_bio.dmard_** | Joint Synovectomized  (P=0.50) | Joint Synovectomized  (P=0.36) | Joint Synovectomized  (P=0.30) |  |
| **(Approx. Spearman:**  **Rho=0.56)**  **N= 11, obs. =28** | Synovectomy position  (P=0.97) |  |  |  |
|  | Side  (P=0.78) | Side  (P=0.79) |  |  |
|  | **Δ**BME  (P=0.07) | **Δ**BME  (P=0.02) | **Δ**BME  (P=0.02) | **Δ**BME  (P=0.01) |
| √(MCP-1**_Isotype control_)** | Joint Synovectomized  (P=0.18) | Joint Synovectomized  (P=0.16) | Joint Synovectomized  (P=0.12) |  |
| **(Approx. Spearman:**  **Rho=0.48)**  **N= 11, obs. =28** | Synovectomy position  (P=0.64 ) | Synovectomy position  (P=0.62 ) |  |  |
|  | Side  (P=0.76) |  |  |  |
|  | **Δ**BME  (P=0.42 ) | **Δ**BME  (P=0.45 ) | **Δ**BME  (P=0.20 ) |  |
|  |  |  |  |  |

This table depicts the statistical associations between the change in RAMRIS BME score in bDMARD treated RA patients (N=11, 28 explants) and change in synovial explant mediator release after 2 weeks of culture. A mixed model has been used for the statistical analysis, P<0.05 was considered significant. In the reduced model covariates were excluded if P>0.10. All of the four pre-specified covariates, tested in the models, are illustrated above.

bDMARD = biologic disease modifying anti-rheumatic drugs; RAMRIS BME= Rheumatoid Arthritis Magnetic Resonance Imaging Score for Bone Marrow Oedema. Log_10_= 10 logarithm, **√ =** square root. Inv = Inverted. *= model control failed normal distribution of residuals.

Covariates included in the statistical model: Joint Synovectomized = Wrist, MCP or PIP; Synovectomy position = Ulnar, central, radial or mixed for pooled synovectomy positions; Side = left or right; MCP-1 = Monocyte Chemoattractant Protein 1; MCP = metacarpophalangeal joint; PIP = Proximal interphalangeal joint.
